# Evaluation of text-mining systems for biology: overview of the Second BioCreative community challenge

**DOI:** 10.1186/gb-2008-9-s2-s1

**Published:** 2008-09-01

**Authors:** Martin Krallinger, Alexander Morgan, Larry Smith, Florian Leitner, Lorraine Tanabe, John Wilbur, Lynette Hirschman, Alfonso Valencia

**Affiliations:** 1 Structural Biology and BioComputing Programme, Spanish Nacional Cancer Research Centre (CNIO), C/Melchor F. Almagro, 3, E-28029 Madrid, Spain; 2 Biomedical Informatics, Stanford University, Stanford, California 94306, USA; 3 National Center for Biotechnology Information, 8600 Rockville Pike, Bethesda, Maryland, 20894, USA; 4 Information Technology Center, The MITRE Corporation, 202 Burlington Road, Bedford, Massachusetts 01730, USA

## Abstract

**Background:**

Genome sciences have experienced an increasing demand for efficient text-processing tools that can extract biologically relevant information from the growing amount of published literature. In response, a range of text-mining and information-extraction tools have recently been developed specifically for the biological domain. Such tools are only useful if they are designed to meet real-life tasks and if their performance can be estimated and compared. The BioCreative challenge (Critical Assessment of Information Extraction in Biology) consists of a collaborative initiative to provide a common evaluation framework for monitoring and assessing the state-of-the-art of text-mining systems applied to biologically relevant problems.

**Results:**

The Second BioCreative assessment (2006 to 2007) attracted 44 teams from 13 countries worldwide, with the aim of evaluating current information-extraction/text-mining technologies developed for one or more of the three tasks defined for this challenge evaluation. These tasks included the recognition of gene mentions in abstracts (gene mention task); the extraction of a list of unique identifiers for human genes mentioned in abstracts (gene normalization task); and finally the extraction of physical protein-protein interaction annotation-relevant information (protein-protein interaction task). The 'gold standard' data used for evaluating submissions for the third task was provided by the interaction databases MINT (Molecular Interaction Database) and IntAct.

**Conclusion:**

The Second BioCreative assessment almost doubled the number of participants for each individual task when compared with the first BioCreative assessment. An overall improvement in terms of balanced precision and recall was observed for the best submissions for the gene mention (F score 0.87); for the gene normalization task, the best results were comparable (F score 0.81) compared with results obtained for similar tasks posed at the first BioCreative challenge. In case of the protein-protein interaction task, the importance and difficulties of experimentally confirmed annotation extraction from full-text articles were explored, yielding different results depending on the step of the annotation extraction workflow. A common characteristic observed in all three tasks was that the combination of system outputs could yield better results than any single system. Finally, the development of the first text-mining meta-server was promoted within the context of this community challenge.

## Background

Modern biology increasingly depends on the availability of computational tools to process, analyze, interpret, and integrate large collections of heterogeneous data. Bioinformatics systems are now routinely used for tasks such as analysis of genome sequences, protein structures, or interactions derived from publicly accessible databases. A considerable fraction of the existing data in biology consists of natural language texts used to describe and communicate new discoveries. Scientific papers constitute a resource with crucial importance for life sciences and the literature database PubMed stores around 16 million citations and receives over 70 million queries every month.

It is not just the general biology user who needs more efficient access to specific information contained in article collections; the literature also constitutes the primary knowledge resource that database curators use to derive manual annotations. This implies that most of the current functional information contained in biological databases has been extracted directly or indirectly from articles.

A range of recently implemented text mining strategies is currently available to facilitate more sophisticated biomedical literature processing. A survey of some of the most relevant applications can be found in this supplement to *Genome Biology *[1].

From the user perspective, it is often cumbersome to decide which application is the most suitable for a given problem without a proper benchmark study comparing their performance on a common data collection. An analogous scenario is encountered for experimental characterizations, in which alternative technologies can yield comparable results. A range of independent benchmark studies have been published comparing different bioinformatics systems, for instance for the prediction of signal peptides [2] or subcellular localization prediction methods [3]. Although these studies are practically useful for certain scenarios, they are often only suitable for very specific applications with no directly comparable results. Community challenges constitute an alternative strategy, which promote the development of tools assessed and compared on the basis of an impartial evaluation based on a common dataset. These are not only useful to determine the state-of-the-art in a certain field, based on specified tasks, but they also serve as a way to monitor improvements over time. In general, community assessments address the following issues:

1. Comparison of different methods and strategies on a common task.

2. Determination of the state of the art of a field.

3. Monitoring of improvements in the field.

4. Ability of the technology to meet real world needs.

5. Development of useful 'gold standard' data collections.

6. Exploration of meaningful evaluation strategies.

7. Promotion of community-based collaborative effort.

**Figure 1:**
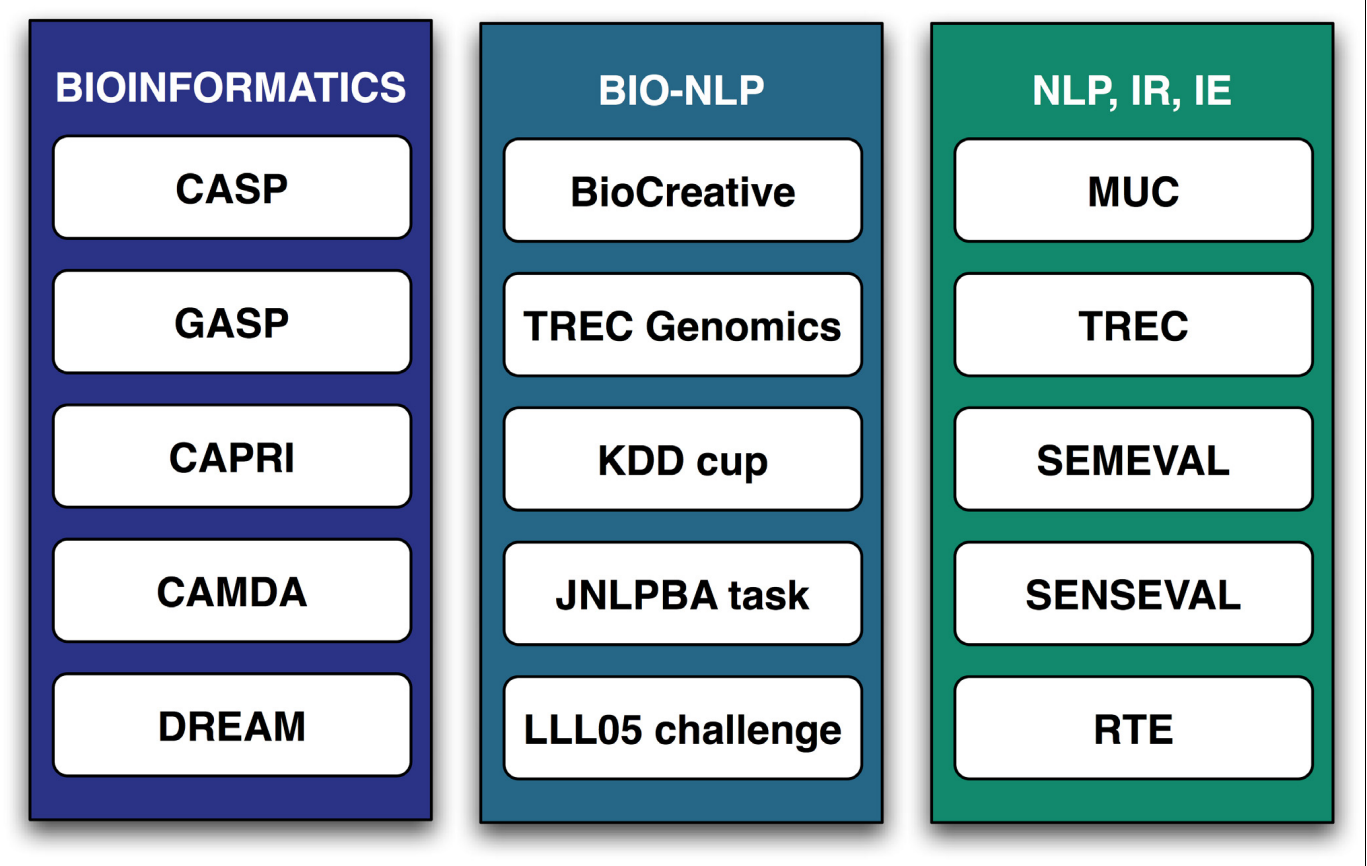
Community evaluations: from bioinformatics to NLP. CAMDA, Critical Assessment of Microarray Data Analysis; CAPRI, Critical Assessment of PRediction of Interactions; CASP, Critical Assessment of Techniques for Protein Structure Prediction; GASP, Genome Annotation Assessment Project; IE, information extraction; IR, information retrieval; JNLPBA, Joint Workshop on Natural Language Processing in Biomedicine and its Applications; KDD, Knowledge Discovery and Data Mining; LLL, Learning Language in Logic; MUC, Message Understanding Conference; NLP, natural language processing; DREAM, Dialogue on Reverse Engineering Assessment and Methods; RTE, Recognising Textual Entailment Challenge; SEMEVAL, Semantic Evaluations; SENSEVAL, Evaluating Word Sense Disambiguation Systems; TREC, Text Retrieval Conference.

Figure 1 provides an overview of some of the most relevant assessments that have been carried out in the bioinformatics and text-mining domains. Not all of the community challenges focus on determining the state-of-the-art of existing technologies. For instance, in the field of protein engineering, the aim of the Paracelsus challenge was to determine whether it was possible to transform the conformation of a globular protein into another by altering no more than half of the sequence [4].

Bioinformatics assessments are generally more concerned with comparing results from different computational strategies against experimentally generated data collections, such as protein structures resolved using x-ray crystallography or biochemically determined functional sites. For example, in the Critical Assessment of Techniques for Protein Structure Prediction (CASP) competition, the overall aim is to evaluate structural bioinformatics tools by comparing automatically predicted protein models based on sequence with the experimentally solved protein structures [5]. In case of the Genome Annotation Assessment Project (GASP) the goal was to score the performance of tools for finding protein coding genes and to determine the accuracy of automatic genome annotation systems [6].

A very active area of research has been the evaluation of strategies for automatically processing natural language by means of computational tools. The Message Understanding Conferences (MUCs) served as a framework to promote research in the information extraction domain, posing tasks such as the recognition of entity names or temporal expressions in text [7]. The Recognising Textual Entailment Challenge (RTE) [8] focused on inference, and whether one text was 'entailed' by (could be inferred from) another.

Reflecting the importance of literature data for biology, there have been several evaluations for text-mining and information retrieval strategies specifically for this domain. A Knowledge Discovery and Data Mining (KDD) Challenge Cup task in 2002 [9] focused on prioritizing articles for curation in FlyBase, based on presence of experimental evidence for gene expression in the articles. One of the main goals of the Text Retrieval Conference (TREC) Genomics track was to evaluate information retrieval and question answering systems for biomedical topics [10]. Other assessments have included the Genic Interaction Extraction Challenge (LLL05) for extracting genetic interactions from PubMed abstracts [11] and the Bio-Entity Recognition task of the BioNLP/NLPBA workshop, which is concerned with the detection of concepts of biological interest, such as genes and proteins [12].

### BioCreative

The BioCreative (Critical Assessment of Information Extraction systems in Biology) challenge evaluation is a community-wide effort for the evaluation of text-mining and information extraction systems applied to the biological domain. The goal of BioCreative has been to pose tasks that will result in systems that can scale for use by general biology researchers or more specialized end users such as annotation database curators. An important contribution of BioCreative has been the creation of shared gold standard datasets, prepared by domain experts, for the training and testing of text mining applications. These collections (and the associated evaluation methods) represent an important resource for continued development and improvement of text-mining applications.

### BioCreative I

The first BioCreative challenge evaluation, carried out during 2003 to 2004, was organized through collaborations between different groups from the text-mining, biological database, and bioinformatics domains [13]. Participants had to provide submissions for two tasks; the first one related to the identification of gene mentions in text (task 1A) [14] and linking gene database entries to PubMed abstracts (task 1B) [15]. The second assignment was related to the extraction of human gene products with annotations of Gene Ontology terms supported by textual evidence passages [16].

### BioCreative II

**Figure 2:**
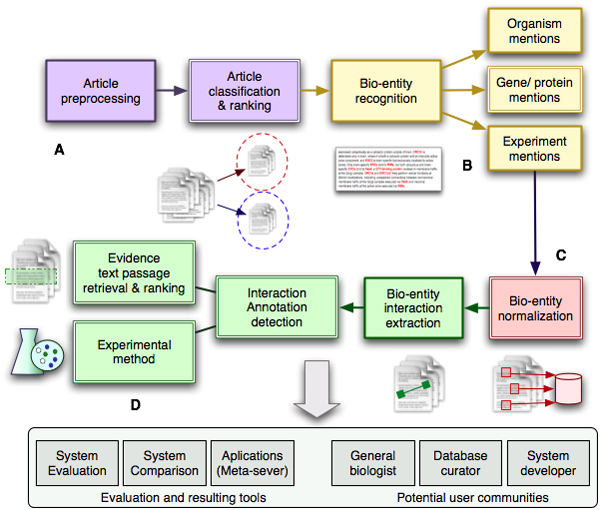
BioCreative II tasks. This figure illustrates the basic processing steps covered by the tasks and subtasks posed in BioCreative II. Note that not all of the data collections were aligned (the gene mention [GM], gene normalization [GN], and protein-protein interaction [PPI] tasks used different document collections). **(A) **Preprocessing of full-text articles was provided in different commonly available formats including HTML, PDF, and automatic plain text conversions from these formats was covered by the interaction pair subtask (IPS), interaction method subtask (IMS), and interaction sentences subtask (ISS). The detection and ranking of abstracts relevant for a given biological topic (in this case protein-protein interactions) was part of the interaction article subtask (IAS). **(B) **Labeling text with bio-entities of interest was part of the GM task, in which participants had to find gene and protein mentions automatically. **(C) **To provide direct links of abstracts and full-text articles to database entries, a process often called protein or gene normalization was part of the GN and IPS tasks, respectively. **(D) **Extraction of specific biological relation types (physical protein-protein interactions) was addressed in the IPS, together with the detection of experimental interaction detection methods used for characterizing these interactions. For human interpretation, retrieval of evidence passages summarizing a particular biological association is crucial. This aspect was addressed in the ISS. Different participating systems were evaluated and compared based on test data collections released by the BioCreative II organizers. To allow integration of different strategies, the BioCreative MetaServer (BCMS) was developed.

Because of strong interest in the results of first BioCreative, a second BioCreative challenge was organized during the period from 2006 to 2007 with three tasks. The gene mention (GM) task (similar to the first BioCreative task 1A) focused on the identification of gene and protein names in PubMed abstracts. The gene normalization (GN) task (similar to task 1B in the first BioCreative) evaluated linkage of abstracts to biological database records, focused on listing EntrezGene identifiers for human genes and proteins mentioned in abstracts. Finally, the protein-protein interaction (PPI) task assessed the performance of text-mining systems for extracting from the literature evidence of protein-protein interactions suitable for database curation (figure 2 shows a general overview of the posed tasks).

For each task, a training data collection was made available to allow system development by the participating groups. After a training period, registered participants obtained a test dataset, for which they had to submit results within a relatively short period of time (to avoid any manual result generation). The participating teams provided their automatically generated test set submissions for evaluation. These submissions were then scored against the gold standard data produced by human annotators; these manual annotations were kept blind (were not available) to the participating teams before the submission deadline, to guarantee fair assessment. The agreement between human experts (inter-annotator agreement) was assessed [17] to estimate the difficulty of the GN task. Each participating team could submit up to three runs for any of the subtasks. For the GM and GN tasks, we examined relative improvement with respect to the previous BioCreative. For these tasks, we also estimated that combining multiple participant submissions into a single consensus prediction could improve the overall performance. A question of practical importance is the availability of online text-mining meta-server resources, especially ones that can integrate the consensus output from several online systems. A major outcome of the BioCreative II challenge is that the Spanish National Cancer Centre (CNIO) has hosted the first text-mining meta-server for biology, integrating systems and/or services made available by BioCreative participants. The BioCreative Metaserver can be accessed at: .

## Results

### Gene mention task

A total of 21 teams participated in the GM task of BioCreative II. This task was coordinated by John Wilbur, Larry Smith, and Lorrie Tanabe from the National Center for Biotechnology Information (NCBI). Its goal was to evaluate systems that find mentions of genes and proteins in sentences from PubMed abstracts. The training corpus was based on sentences manually labeled with gene and protein mentions from the training and test data collections of task 1A (BioCreative I). The test data for BioCreative II consisted of a total of 5,000 new blind sentences, for which the participating teams had to provide the substrings corresponding to gene or protein names. The submissions were evaluated against the manually labeled mentions, and scored on the basis of precision, recall, and F score (balanced precision and recall). Overall, the results improved with respect to comparable results from BioCreative task 1A; the highest F measure went from 0.822 in task 1A of BioCreative I to 0.872 in BioCreative II. This difference is significant, provided that one assumes that the changes to the annotations of the corpus made between BioCreative I and II have had no effect on performance. In a series of experiments, the organizers also estimated system performance of various weighted combinations of the participating systems. The best composite system achieved an F measure of 0.905; this system included the results from all 21 systems and demonstrated that even submissions from low-performing systems contributed to increased performance of the composite system [18].

### Gene normalization task

The GN task was organized by Alex Morgan (MITRE/Stanford University) and Lynette Hirschman (MITRE), with 20 teams participating. The task focused on providing direct links between text (PubMed abstracts) and actual gene and protein records in existing biological databases, which is a crucial step in annotation extraction. This task was similar in design to task 1B in the first BioCreative, with the difference that in BioCreative II the focus was on human genes/proteins, whereas for the first BioCreative the linking was done for three model organisms (yeast, mouse, and fly). The task required that, for each PubMed abstract, a participating system return the list of unique EntrezGene identifiers of human genes or proteins mentioned in the abstract. The organizers provided, as training data, a collection of 281 PubMed abstracts manually mapped to human EntrezGene identifiers. The blind test corpus consisted of 261 abstracts manually annotated and then carefully reviewed. The best result obtained for the human gene normalization task was an F score of 0.81, which was comparable to the scores for mouse (0.82) and fly (0.79) from BioCreative I task 1B. In line with the GM task, a composite system created by weighting contributions of all participating systems performed higher than any single system, yielding an F score of 0.92 [17].

### Protein-protein interaction task

The aim of the PPI task was to evaluate the performance of text-mining tools on the automatic extraction of physical protein-protein interaction annotations from the literature, as compared with manual annotations generated by interaction database curators that were used as a gold standard. Martin Krallinger and Alfonso Valencia from CNIO co-ordinated this task, whereas the expert curated training and test data were provided by two extensively used protein-protein interaction databases: the Molecular Interaction Database (MINT) and the IntAct database [19]. In general, annotations entered into these databases require the presence in a publication of explicit experimental evidence supporting a given biological association. Both interaction databases used a common annotation standard, namely the Proteomics Standards Initiative Molecular Interaction (PSI-MI) data exchange format. Both databases also rely on experienced domain experts for extracting manual curations from the literature and conduct exhaustive curation of a collection of journals. Inspired by the manual curation workflow, the PPI task was structured into four subtasks, each focusing on a particular aspect of the underlying curation pipeline. A total of 26 different teams participated in one or more of the PPI subtasks.

#### Interaction article subtask

The interaction article subtask (IAS) addressed the first step in many biology literature review tasks, namely the retrieval/classification and ranking of relevant articles according to a given topic of interest. The aim here was to classify a collection of PubMed titles and abstracts based on their relevance for the derivation of protein-protein interaction annotations. A total of 19 distinct teams participated in this task. The training data consisted of a set of labeled PubMed records; 3,536 were labeled as relevant for protein interaction annotation based on the fact that their corresponding full-text articles had been used to derive annotations by MINT and IntAct; for negative examples, a set of 1,959 abstracts was used that had been judged as not containing relevant information by the IntAct or MINT teams. The test set consisted of 338 relevant abstracts and 339 nonrelevant abstracts from the recent curation activities of the IntAct and MINT groups. The highest scoring systems achieved an F score of 0.78. This is comparable to results from an earlier KDD Challenge Cup task for ranking articles for Flybase curation, which also reported an F measure of 0.78 [9].

#### Interaction pair subtask

The interaction pair subtask (IPS) required the extraction of a nonredundant list of binary protein-protein interaction pairs for each given full-text article. Each interactor protein had to be identified in terms of its corresponding SwissProt database identifier. Only those interaction pairs for which an experimental characterization was described in the article were considered correct (annotation relevant). The training data consisted of the annotations from a set of 740 documents previously curated by either MINT or IntAct. The blind test set comprised a total of 358 full-text documents curated by MINT or IntAct and withheld from release until after the BioCreative evaluation.

Of the 16 teams providing submissions for the IPS, the highest scoring system achieved a precision of 0.39, recall of 0.31, and a balanced F score (macro-averaged) of 0.29. A test subset using only articles containing exclusively SwissProt identifiers (eliminating articles containing interactor proteins with provisional identifiers found only in Translated EMBL [TrEMBL]) made recall somewhat easier, marginally improving the F score; the best performance using only SwissProt was precision of 0.37, recall of 0.33, and a macro-averaged balanced F-score of 0.30.

The interactor protein normalization task turned out to be much more difficult than the GN task; the best scoring team obtained an F measure of 0.52, as compared with around 0.8 for human gene normalization. It is clear that performance on multispecies protein normalization was a major technical obstacle for the IPS. Interestingly, because each interaction required the association of a pair of interacting proteins, the F measure of 0.3 was perhaps slightly higher than would have been expected.

#### Interaction sentences subtask

The interaction sentences subtask (ISS) required the identification of the best evidence passage (up to three sentences) describing each experimentally characterized interaction mentioned in a full-text paper. To create the gold standard, the curators from MINT and IntAct identified the most informative passage for each interaction from the full-text article, as part of their curation of the final test set. Participants could nominate up to five (ranked) passages for each extracted interaction. Results were scored by string comparison (using a sliding window) between the passages selected by the curator against those nominated by each system. Of the 11 participants, the most accurate strategy nominated 361 passages, of which 70 (17%) corresponded to curated ones. The highest recall system nominated 7,526 (ranked) passages of which 343 were correct. However, these scores are artificially low since some of the system nominated passages contained useful information even when they were not judged to be the most informative passage.

#### Interaction method subtask

Knowledge of the experimental methodology supporting a given biological interaction is of particular practical relevance because it provides a qualifier for the associated evidence. The two teams submitting results for the interaction method subtask (IMS) had to identify the experimental method(s) used to determine the protein-protein interactions described in the test set articles. An interaction detection method had to be mapped to its corresponding Molecular Interaction ontology code. Two scores were computed to evaluate the performance of the interaction method association extraction: exact matching to the ontology code and 'parent matching', which scored a match as correct if the submitted code was an exact match to the gold standard term or to its parent term in the Molecular Interaction ontology. The high F score for parent matching was an encouraging 65%; for exact match, the high score was 48%.

## Discussion and conclusion

The Second BioCreative community challenge evaluation successfully promoted the development, evaluation, and monitoring of text-mining strategies applied to biologically relevant tasks. This is reflected in the significant increase in number of participating teams with respect to BioCreative I, as well as in the collaboration with biological databases for providing useful data collections.

The BioCreative initiative is built upon a collaborative effort among researchers from heterogeneous domains, including biology, bioinformatics, and natural language processing. As a result, it has served as a common ground to exchange different views across these domains, making it possible to explore alternative approaches to biologically important tasks which can be approached by text-mining tools. Each of the tasks or subtasks was motivated by a series of practical applications; the analysis of the systems and their performance on these tasks has provided insights into the difficulty of the tasks as well as useful strategies to handle the tasks.

The GM task is important as a component for literature retrieval systems, as well as a component of gene normalization tools (for example, in the GN task). Moreover, collection of new gene names can help to complete and extend gene and protein name collections in existing manually curated databases, especially for organisms whose genomes lack extensive manual support. One of the new features introduced for the BioCreative II GM task was recognition of gene mentions at the character level instead of the word token level, which facilitated the use of alternative word tokenization approaches or even recognition at the substring level instead of tokens. This is especially important when taking into account the inconsistent use of hyphens or other special characters in biomedical texts, which makes robust text tokenization difficult for this domain. Successful approaches to the gene mention task were based on machine learning techniques, particularly conditional random fields (CRFs). There was an overall improvement with respect to the results of BioCreative I, with the performance of the composite system approaching that of equivalent tasks in other domains, such as newswire texts. One issue that still remains unclear is whether a similar performance could be expected for full-text articles.

The importance of the GN task can be conceived from different viewpoints. The task supports a direct connection between mentions of genes and proteins within their textual context in the literature, through a unique database identifier, to sequence information in those databases. Therefore gene normalization tools are essential to link existing online literature repositories to biological databases, one of the main concerns of Semantic Web and data integration technologies for the biological domain. Gene and protein normalization is also a crucial first step toward the extraction of text-based annotations for these entities, such as their associations to controlled vocabulary concepts in ontologies such as Gene Ontology, as well as identification of protein interactions. The results of this task are encouraging, and by combining the various submissions using majority voting, an even better performance can be achieved. The results indicate that human genes are not particularly more difficult to normalize than mouse or fly genes, and that EntrezGene can be used effectively as a reference database. One of the limitations of the GN task design was that normalization was performed on abstracts restricted to human genes. This restriction resulted in an artificial scenario, because in practice the disambiguation and linkage of genes from different species, especially mouse and human genes, is required for real applications, such as seen in the interactor protein normalization task. Nevertheless, by using this controlled set-up, it has been possible to tease apart important aspects of gene normalization performance. This will inform our design of new tasks for subsequent evaluations.

The PPI task demonstrated the usefulness of abstracts for the initial selection of annotation relevant articles, but also that - for the extraction of actual annotation associations - full-text articles are essential. Both the ranking of relevant articles as well as the agreement between different systems may result in article prioritization systems that support more robust curation pipelines. For the development of such document categorization tools serving as annotation support, several aspects can affect performance: the curation strategy underlying the training and test article selection, the journal composition, language change over time (publication dates), and even abstract length. It also remains unclear how the implementations used for BioCreative II would scale up when applied to the whole PubMed database, which also hosts articles from other scientific disciplines.

To support the development of text-mining systems, it would be beneficial to the research community for database maintainers to record and make public additional information arising from the curation process, for example the set of articles reviewed and judged not relevant for curation, and the specific passages within a publication used by a curator to support an annotation.

There also remain important hurdles to the development and use of full-text processing tools, starting with issues related to article availability, copyright and data distribution, and extending to article format and encoding, which can influence whether efficient text processing is feasible. For the extraction of biological annotations such as protein interaction, gene and protein normalization is a crucial initial step. Several aspects will need to be explored in more detail in the future to improve gene normalization tools applied to full-text articles, starting with the actual choice of the databases that should be used as a reference. Here, both SwissProt as well as EntrezGene are certainly valuable resources, especially for well studied genomes, but neither covers all the proteins (and their names or symbols) described in the literature. To overcome database incompleteness, human database curators often use bioinformatics sequence similarity based intermediate steps for the manual normalization to other resources such as TrEMBL, an aspect that is often neglected when comparing information extraction results to biological annotation databases.

The results of BioCreative II showed that combining multiple systems can improve performance. As a result, BioCreative has spurred the development of the first text-mining meta-server, which will serve as a framework and platform to improve the accessibility and use of automatically extracted text-derived information by the user community. The server delivers to the user consensus annotations for PubMed abstracts from systems that participated in the BioCreative II challenge. It also allows side-by-side comparison of different participant systems. It will be interesting to use these consensus annotations as a baseline for future BioCreative challenges. Furthermore, other research groups can add to the platform, providing their own annotations - including new annotation types - or can use its output for their own purposes. One of the limitations in the set of BioCreative tasks was that different tasks were not aligned in terms of the data collections used. Future improvements would include the use of a common dataset for all three tasks, evaluating the gene mention, gene normalization, and biological annotation extraction on the same set of articles.

This BioCreative II supplement to *Genome Biology *includes the following: articles devoted to overviews of each of the three tasks; papers from a select set of participating teams who achieved good performance on multiple tasks or subtasks of BioCreative II; a review article providing an overview and introduction to existing text-mining systems for the biological domain; an article describing the BioCreative II meta-server; and an opinion paper consisting of multiple views on the current state-of-the-art and future prospects for text mining in biology.

## Materials and methods

### Data and corpus collections

A detailed description of the data preparation for each task can be found in the corresponding task overview articles within this supplement to *Genome Biology*. For the GM task, the training data consisted of the 15,000 sentences used in the first BioCreative (task 1A), with some additional revisions to improve the annotation consistency and to allow gene mention finding on the character level rather than on the word token level. The GM test data consisted of 5,000 additional PubMed sentences.

The GN task used similar procedures to those used in the first BioCreative 1B task. Citations derived from Gene Ontology Annotation records were used to select the documents. Domain experts extracted links of the human genes mentioned in these abstracts to their corresponding EntrezGene identifiers. To check annotation consistency, a human inter-annotator agreement experiment was performed, with results of around 90% pair-wise agreement. The final data collection provided for this task consisted of 281 abstracts for training and 262 for testing.

For the PPI task, the MINT and IntAct interaction database provided the data collections. For both databases, trained domain experts manually extract relevant interaction information from the literature to fill in structured database records, after detailed reading of full text articles. In general, there are three basic strategies for selection of articles for annotation: recommendation-based curation, where annotated article citations are mainly derived from other resources or databases; exhaustive curation of a specific set of journals of interest, where each article is examined in detail to derive potential annotations; and thematic curation, where articles are selected primarily based on a keyword search (for example, for cancer or cell cycle related papers). The IAS training data consisted of 3,536 protein abstracts that had been judged to be relevant for interaction annotation (according to any of the selection strategies), based on the actual content of both databases. Additionally, a set of 1,959 negative examples was distributed, corresponding to abstracts that were found not to be relevant to protein interaction annotation on the basis of exhaustive curation. The IAS test set had a total of 338 interaction relevant and 339 nonrelevant, recently published abstracts, generated by exhaustive curation. For the remaining PPI subtasks, the training data consisted of a set of 740 full-text documents previously curated by either MINT or IntAct, together with the corresponding annotation record. Each article was distributed in various formats, including PDF and HTML, as well as automatically generated plain text conversions. The blind test set consisted of 358 full-text documents curated by MINT or IntAct; these were withheld from release until after the BioCreative evaluation.

### Overview of methods of participating systems

The participating teams employed a variety of methods to produce submissions for BioCreative II tasks, ranging from machine learning techniques such as support vector machines [20] or CRFs [21], to approximate string matching and manually generated rules and patterns.

For detecting gene mentions, many teams used CRFs, part-of-speech tagging, and stemming. Some participants also exploited domain-specific systems such as the GENIA tagger [22] or adapted general natural language processing (NLP) tools such as Mallet [23].

In case of the GN task, many groups used dictionary look-up methods, often coupled with sophisticated dictionary expansion and/or editing methods on existing databases such as EntrezGene. Other techniques included generation of lexical variants for gene and protein names and the filtering of highly ambiguous symbols. A number of systems applied sophisticated approximate string matching strategies and several used context-based disambiguation of gene names. Several teams combined one or more gene mention systems, similar to those used for the GM task, with a dictionary look-up approach to produce the ranked list of EntrezGene identifiers.

For the classification of interaction-relevant articles, the vast majority of systems used machine learning techniques, with support vector machines being the most successful approach. For the detection of the interaction pairs, most of the systems assumed co-occurrence of the interactor proteins within a predefined unit of text, often within a sentence. Nevertheless, some participants also took into account additional aspects, such as the average distance between the protein mentions derived from the whole paper. Overall, the strategies included the use of supervised learning-based sentence classifiers; detection of interaction-relevant verbs, keywords or word patterns; rule-based systems; use of information on the relative position of the sentences within the full-text article (for example, text from figure legends); integration of NLP-related modules such as syntactic parsing; and the handling of coordinated expressions in cases in which more than a single protein pair was mentioned in a given sentence. Given the prevalence of inter-organism protein name ambiguity, a crucial step for the correct normalization of the interactor proteins was the association of proteins with their corresponding organism source, for instance whether they corresponded to mouse or human.

## Abbreviations

CNIO, Spanish National Cancer Centre; CRF, conditional random field; GM, gene mention; GN, gene normalization; IAS, interaction article subtask; IMS, interaction method subtask; IPS, interaction pair subtask; ISS, interaction sentences subtask; NCBI, National Center for Biotechnology Information; NLP, natural language processing; PPI, protein-protein interaction; TrEMBL, Translated EMBL.

## Competing interests

The authors declare that they have no competing interests.

## Authors' contributions

The GN task was organized by AM and LH, and the description of the task was written by LH. The GM task was organized by LS, LT, and JW. The PPI task was organized by MK, FL, and AV. The description of the PPI task and general sections of the introductory paper were initially written by MK with contributions from LH, JW, and FL.
